# Morbidity prediction in conservatively managed rib fracture patients

**DOI:** 10.1007/s00068-025-02860-4

**Published:** 2025-04-29

**Authors:** Lovisa Ekestubbe, Maximilian Peter Forssten, Yang Cao, Babak Sarani, Shahin Mohseni

**Affiliations:** 1 Department of Orthopedic Surgery, Orebro University Hospital, Orebro, Sweden; 2 School of Medical Sciences, Orebro University, Orebro, Sweden; 3 Clinical Epidemiology and Biostatistics, School of Medical Sciences, Faculty of Medicine and Health, Orebro University, Orebro, Sweden; 4 Center of Trauma and Critical Care, George Washington University, Washington, DC USA

**Keywords:** Rib fracture, Conservative management, Morbidity, Permutation importance, Machine learning

## Abstract

**Purpose:**

Rib fractures, common in blunt chest trauma, affect 10% of trauma patients and are linked to increased pulmonary morbidity and mortality. This study applies machine learning to identify predictors of complications in conservatively managed rib fracture patients.

**Methods:**

Data from the 2013–2021 American College of Surgeons’ Trauma Quality Improvement Program included adults (≥ 18 years) with isolated thoracic injury from blunt trauma and conservatively managed rib fractures. Variables included demographics, comorbidities, injury severity, injury patterns, admission vitals, and complications. The permutation importance method identified top predictors of in-hospital complications.

**Results:**

Of 321,355 rib fracture patients, 183,303 (57.0%) had isolated rib fractures. The five primary predictors of complications in all rib fracture patients were age, Glasgow Coma Scale (GCS) on admission, Revised Cardiac Risk Index (RCRI), chronic obstructive pulmonary disease (COPD), and alcohol use disorder. For isolated rib fracture patients, the same predictors applied but in the order: age, RCRI, GCS, COPD, and alcohol use disorder. A logistic regression model using these predictors showed acceptable discriminative capacity for complications in the full cohort [AUC (95% CI): 0.72 (0.71–0.72)] and isolated rib fracture patients [AUC (95% CI): 0.72 (0.71–0.73)].

**Conclusion:**

Cardiovascular risk, age, and level of consciousness on admission are key predictors of complications in conservatively managed rib fracture patients. Though complication rates remain low overall, elderly patients with multiple cardiovascular risk factors face a heightened risk of deterioration.

**Supplementary Information:**

The online version contains supplementary material available at 10.1007/s00068-025-02860-4.

## Introduction

In the realm of trauma care, injuries to the thoracic cage and its contents are pervasive, resulting in morbidity and mortality as well as shaping critical care approaches for a multitude of patients [1–3]. The challenge lies in the unpredictability of further decompensation and loss of chest wall function, making the identification of patients prone to subsequent decompensation difficult [3–5]. Rib fractures are present in approximately 10% of all trauma patients and 50% of those who have suffered blunt chest trauma [6]. Furthermore, rib fractures are linked to higher mortality rates and severe pulmonary morbidity [6, 7].

Accordingly, finding a way to prognosticate adverse outcomes in patients with rib fractures has received significant interest. Nevertheless, the validation of these scores outside of their originating institutions has been limited [4, 8–13]. Furthermore, while many individual risk factors for patient deterioration have been identified [13–16], the value they provide in predicting adverse outcomes in combination has not been as thoroughly investigated. The aim of the current investigation was therefore to leverage machine learning techniques in order to investigate which variables demonstrate the highest feature importance when predicting complications in non-operatively managed rib fracture patients, using a well validated, national dataset. This approach enabled the identification of key variables most useful for predicting complications, streamlining bedside decision-making by reducing the number of factors to consider. The hypothesis was that in-hospital complications would primarily be predicated upon the condition and characteristics of rib fracture patients on admission.

## Methods

Data were abstracted from the 2013–2021 American College of Surgeons’ Trauma Quality Improvement Program (TQIP) database. Owing to the anonymized, retrospective nature of this dataset, the requirement for ethical approval was waived by the relevant institutional review board. The study complied with the STROBE guidelines and the Declaration of Helsinki [17]. The data obtained from the TQIP database included age, sex, race, comorbidities, abbreviated injury scale (AIS), injury pattern and mechanism of injury, method of analgesia, administered medications, admission vitals, level of trauma center, insurance, as well as complications. All adult patients (age 18 or older) with a conservatively managed rib fracture who suffered an isolated thoracic injury due to blunt trauma were eligible for inclusion. Consequently, patients who underwent any form of thoracic surgery were excluded. Patients with a thorax AIS of 6 were removed as these injuries are in most cases not considered to be survivable. An isolated rib fracture was defined as a thorax AIS ≥ 1 and an AIS ≤ 1 in all other regions.

### Calculating the revised cardiac risk index

The Revised Cardiac Risk Index (RCRI) is composed of six independent, dichotomous variables: a history of ischemic heart disease, congestive heart failure, a history of cerebrovascular disease, diabetes mellitus, renal insufficiency (defined as acute kidney injury or chronic kidney disease), and high-risk surgery. A patient received one point for each variable present. High-risk surgery is defined as any intraperitoneal, intrathoracic, or suprainguinal vascular procedure; however, no points were awarded for this variable since all patients were conservatively managed [8]. 

### Statistical analysis

The primary outcome was in-hospital complications (myocardial infarction, cardiac arrest with CPR, stroke, deep vein thrombosis, pulmonary embolism, acute respiratory distress syndrome, or pneumonia). Patients were divided into cohorts based on whether or not they suffered an in-hospital complication. Normally distributed continuous data were summarized using means and standard deviations and the statistical significance of differences between the cohorts was evaluated using the Student’s t-test. Non-normally distributed continuous data were presented using medians and interquartile ranges and the statistical significance of differences between the cohorts was determined using the Mann-Whitney U-test. Categorical variables were summarized as counts and percentages; the Chi-squared test or Fisher’s exact test were applied, as appropriate, to ascertain the statistical significance of differences between the cohorts.

A logistic regression (LR) model was constructed with in-hospital complications as the response variable while the explanatory variables consisted of age, sex, race, injury severity score (ISS), highest AIS in each region, flail chest, sternum fracture, intrathoracic injuries (pneumothorax, hemothorax, pulmonary contusion), number of rib fractures, use of regional, epidural, or spinal analgesia, RCRI [8], Orthopedic Frailty Score (OFS) [9, 18], shock index, vitals on admission to the emergency room (systolic blood pressure < 90 mmHg, pulse > 100 bpm, temperature < 35ºC, temperature ≥ 38,5ºC, saturation < 90%, respiratory rate > 20, respiratory rate < 12), Glasgow Coma Scale (GCS) on admission to the emergency room, anticoagulant therapy, use of corticosteroids, advanced directives limiting care, trauma center level, insurance status, mechanism of injury, as well as comorbidities (hypertension, history of peripheral vascular disease, dementia, chronic obstructive pulmonary disease, smoking status, cirrhosis, coagulopathy, currently receiving chemotherapy for cancer, metastatic cancer, drug use disorder, alcohol use disorder, and major psychiatric illness).

The relative importance of the explanatory variables for predicting in-hospital complications was based on their permutation importance (PI) [19]. The PI was determined by evaluating how much a specific value [1 - Area under the receiver-operating characteristic curve (AUC)] was modified by the suppression of a particular variable. Instead of merely removing each variable from the dataset, the PI method replaces the variable with noise from other cases by rearranging each variable’s values ​​to conceal the variable’s information during evaluation. This procedure was replicated 10 times to compensate for the random nature of permutations. The relative importance of each variable in the model was calculated as the average increase in 1-AUC compared to the AUC in a model including all variables without any permutations.

After calculating the PI for each variable, an additional LR model was fitted using the top five most important variables for predicting in-hospital complications [20, 21]. The AUC along with the sensitivity, specificity, and accuracy that maximized Youden’s index (sensitivity + specificity – 1) were calculated for this model. The 95% confidence interval (CI) for the AUC was determined using the variance of the AUC as defined by DeLong et al. [22], employing the algorithm described by Sun and Xu [23]. The remaining CIs were estimated using 2000 stratified bootstrap replicates. Calculating the AUC in this manner serves to demonstrate how well the top 5 predictors function as predictors, but it is not intended to develop a model for predicting clinical outcomes. A subgroup analysis on patients with isolated rib fractures was also performed using the same methodology. An isolated rib fracture was defined as a rib fracture without a concomitant pneumothorax, hemothorax, or pulmonary contusion.

Statistical significance was defined as a two-sided p-value less than 0.05. Missing data were managed using multiple imputation by chained equations. This approach was selected as several studies have demonstrated that multiple imputation often outperforms complete case analysis, particularly in datasets with a substantial number of auxiliary variables [24–26]. Analyses were performed using the statistical software R 4.2.2 (R Foundation for Statistical Computing, Vienna, Austria) using the tidyverse, DALEX, pROC, haven, cowplot, mice, and parallel packages [27]. 

## Results

After applying the inclusion and exclusion criteria, 321,355 patients remained for further investigation, of whom 183,303 (57.0%) suffered isolated rib fractures. Patients who suffered a complication had a median age that was 6 years older (70 vs. 64 years old, *p* < 0.001), and where almost twice as likely to be frail (OFS ≥ 2: 8.8% vs. 5.3%, *p* < 0.001). Additionally, patients who suffered a complication were less often female (30.3% vs. 36.2%, *p* < 0.001), and exhibited a higher cardiac risk based their RCRI (RCRI ≥ 2: 12.8% vs. 4.8%, *p* < 0.001). The prevalence of all comorbidities was higher among those who suffered an in-hospital complication (Table 1).

**Table 1:** Demographics of conservatively managed rib fracture patients

	No complication (*N* = 312,217)	Any complication (*N* = 9,138)	*P*-value
Age, median [IQR]	64 [51–76]	70 [59–79]	< 0.001
Sex, n (%)			< 0.001
Female	112,937 (36.2)	2,769 (30.3)	
Male	198,929 (63.7)	6,359 (69.6)	
Missing	351 (0.1)	10 (0.1)	
Race, n (%)			< 0.001
Non-Hispanic White	226,925 (72.7)	7,100 (77.7)	
Hispanic or Latino	11,989 (3.8)	231 (2.5)	
Black	26,117 (8.4)	625 (6.8)	
Other	27,505 (8.8)	590 (6.5)	
Missing	19,681 (6.3)	592 (6.5)	
RCRI, n (%)			< 0.001
0	233,317 (74.7)	5,331 (58.3)	
1	64,278 (20.6)	2,637 (28.9)	
2	12,335 (4.0)	925 (10.1)	
3	2,061 (0.7)	218 (2.4)	
≥4	226 (0.1)	27 (0.3)	
OFS, n (%)			< 0.001
Non-frail (OFS 0)	248,556 (79.6)	6,071 (66.4)	
Pre-frail (OFS 1)	47,084 (15.1)	2,260 (24.7)	
Frail (OFS ≥ 2)	16,577 (5.3)	807 (8.8)	
Hypertension, n (%)	143,439 (45.9)	5,556 (60.8)	< 0.001
History of angina, n (%)	680 (0.2)	41 (0.4)	< 0.001
Previous myocardial infarction, n (%)	3,839 (1.2)	234 (2.6)	< 0.001
Congestive heart failure, n (%)	16,522 (5.3)	1,204 (13.2)	< 0.001
History of peripheral vascular disease, n (%)	3,285 (1.1)	237 (2.6)	< 0.001
Cerebrovascular disease, n (%)	9,881 (3.2)	516 (5.6)	< 0.001
Dementia, n (%)	15,471 (5.0)	612 (6.7)	< 0.001
Non-independent functional status, n (%)	26,632 (8.5)	1,288 (14.1)	< 0.001
Institutionalized, n (%)	7,504 (2.4)	289 (3.2)	< 0.001
Currently receiving chemotherapy for cancer, n (%)	1,605 (0.5)	83 (0.9)	< 0.001
Metastatic cancer, n (%)	2,453 (0.8)	132 (1.4)	< 0.001
History of malignancy, n (%)	3,929 (1.3)	201 (2.2)	< 0.001
COPD, n (%)	35,953 (11.5)	2,534 (27.7)	< 0.001
Current smoker, n (%)	64,989 (20.8)	2,427 (26.6)	< 0.001
Chronic renal failure, n (%)	5,737 (1.8)	394 (4.3)	< 0.001
Diabetes mellitus, n (%)	58,799 (18.8)	2,351 (25.7)	< 0.001
Cirrhosis, n (%)	4,560 (1.5)	364 (4.0)	< 0.001
Coagulopathy, n (%)	11,717 (3.8)	675 (7.4)	< 0.001
Drug use disorder, n (%)	15,609 (5.0)	552 (6.0)	< 0.001
Alcohol use disorder, n (%)	24,407 (7.8)	1,486 (16.3)	< 0.001
Major psychiatric illness, n (%)	35,120 (11.2)	1,247 (13.6)	< 0.001
Advanced directive limiting care, n (%)	10,093 (3.2)	584 (6.4)	< 0.001

RCRI, Revised Cardiac Risk Index; OFS, Orthopedic Frailty Score; COPD, chronic obstructive pulmonary disease

Patients who suffered a complication also tended to be more severely injured (Thorax AIS ≥ 3: 79.2% vs. 64.3%, *p* < 0.001). Consequently, they were more likely to be hypotensive (3.9% vs. 1.3%, *p* < 0.001), tachycardic (26.6% vs. 16.6%, *p* < 0.001), desaturated (9.8% vs. 3.3%, *p* < 0.001), and tachypneic (28.9% vs. 18.5%, *p* < 0.001) on admission. Furthermore, they were more likely to be unconscious at admission (GCS ≤ 8: 7.4% vs. 1.3%, *p* < 0.001). Patients who suffered an in-hospital complication were therefore also more likely to have suffered multiple rib fractures (90.7% vs. 83.0%, *p* < 0.001), a concomitant flail chest (7.5% vs. 2.5%, *p* < 0.001), sternal fracture (7.5% vs. 5.8%, *p* < 0.001), pneumothorax (38.2% vs. 31.3%, *p* < 0.001), hemothorax (26.5% vs. 12.9%, *p* < 0.001), or pulmonary contusion (19.1% vs. 13.9%, *p* < 0.001). The use of regional (3.2% vs. 1.4%, *p* < 0.001), epidural (1.7% vs. 0.5%, *p* < 0.001), and spinal analgesia (1.6% vs. 0.5%, *p* < 0.001) was more common among those who suffered a complication (Table 2).

**Table 2:** Clinical characteristics of conservatively managed rib fracture patients

	No complication (*N* = 312,217)	Any complication (*N* = 9,138)	*P*-value
Injury Severity Score, median [IQR]	9.0 [5.0–10]	9.0 [9.0–10]	< 0.001
Head AIS, n (%)			0.373
Injury not present	276,927 (88.7)	8,133 (89.0)	
1	35,290 (11.3)	1,005 (11.0)	
Face AIS, n (%)			0.651
Injury not present	269,351 (86.3)	7,899 (86.4)	
1	42,866 (13.7)	1,239 (13.6)	
Neck AIS, n (%)			0.307
Injury not present	309,314 (99.1)	9,043 (99.0)	
1	2,903 (0.9)	95 (1.0)	
Spine AIS, n (%)			0.006
Injury not present	308,954 (99.0)	9,069 (99.2)	
1	3,263 (1.0)	69 (0.8)	
Thorax AIS, n (%)			< 0.001
1	31,953 (10.2)	494 (5.4)	
2	79,569 (25.5)	1,409 (15.4)	
3	186,608 (59.8)	6,190 (67.7)	
4	12,457 (4.0)	875 (9.6)	
5	1,630 (0.5)	170 (1.9)	
Abdomen AIS, n (%)			< 0.001
Injury not present	287,542 (92.1)	8,276 (90.6)	
1	24,675 (7.9)	862 (9.4)	
Upper extremity AIS, n (%)			0.930
Injury not present	256,380 (82.1)	7,500 (82.1)	
1	55,837 (17.9)	1,638 (17.9)	
Lower extremity AIS, n (%)			0.409
Injury not present	263,751 (84.5)	7,749 (84.8)	
1	48,466 (15.5)	1,389 (15.2)	
External/Other AIS, n (%)			0.036
Injury not present	296,141 (94.9)	8,622 (94.4)	
1	16,076 (5.1)	516 (5.6)	
Flail chest, n (%)	7,898 (2.5)	687 (7.5)	< 0.001
Sternal fracture, n (%)	18,066 (5.8)	689 (7.5)	< 0.001
Pneumothorax, n (%)	97,776 (31.3)	3,495 (38.2)	< 0.001
Hemothorax, n (%)	40,144 (12.9)	2,426 (26.5)	< 0.001
Pulmonary contusion, n (%)	43,406 (13.9)	1,745 (19.1)	< 0.001
Number of rib fractures, n (%)			< 0.001
Single	53,147 (17.0)	852 (9.3)	
Multiple	259,070 (83.0)	8,286 (90.7)	
Method of analgesia, n (%)			
Regional	4,486 (1.4)	288 (3.2)	< 0.001
Epidural	1,588 (0.5)	153 (1.7)	< 0.001
Spinal	1,629 (0.5)	146 (1.6)	< 0.001
Steroid use, n (%)	4,246 (1.4)	272 (3.0)	< 0.001
Anticoagulant therapy, n (%)	1,767 (0.6)	29 (0.3)	0.003
Missing	47,381 (15.2)	1,507 (16.5)	
Shock index, median [IQR]	0.59 [0.49–0.70]	0.63 [0.51–0.78]	< 0.001
Missing, n (%)	9172 (2.9)	364 (4.0)	
Systolic blood pressure < 90 mmHg, n (%)	4,035 (1.3)	354 (3.9)	< 0.001
Missing	7,441 (2.4)	260 (2.8)	
Pulse rate > 100 bpm, n (%)	51,887 (16.6)	2,429 (26.6)	< 0.001
Missing	6,896 (2.2)	233 (2.5)	
Temperature < 35 °C, n (%)	1,232 (0.4)	127 (1.4)	< 0.001
Missing	24,585 (7.9)	1,008 (11.0)	
Temperature ≥ 38 °C, n (%)	1,969 (0.6)	122 (1.3)	< 0.001
Missing	24,585 (7.9)	1,008 (11.0)	
Saturation < 90%, n (%)	10,284 (3.3)	891 (9.8)	< 0.001
Missing	14,064 (4.5)	535 (5.9)	
Respiratory rate > 20, n (%)	57,908 (18.5)	2,641 (28.9)	< 0.001
Missing	9,267 (3.0)	388 (4.2)	
Respiratory rate < 12, n (%)	3,079 (1.0)	192 (2.1)	< 0.001
Missing	9,267 (3.0)	388 (4.2)	
GCS on admission, n (%)			< 0.001
Mild (GCS 14–15)	283,837 (90.9)	7,540 (82.5)	
Moderate (GCS 9–13)	4,981 (1.6)	337 (3.7)	
Severe (GCS 3–8)	4,204 (1.3)	674 (7.4)	
Missing	19,195 (6.1)	587 (6.4)	
Trauma center level, n (%)			< 0.001
Level 1	114,747 (36.8)	3,801 (41.6)	
Level 2	79,591 (25.5)	2,197 (24.0)	
Level 3	21,319 (6.8)	454 (5.0)	
Not verified/designated	96,560 (30.9)	2,686 (29.4)	
Insurance, n (%)			< 0.001
Commercial insurance	106,462 (34.1)	2,306 (25.2)	
Non-commercial insurance	164,280 (52.6)	5,943 (65.0)	
Self-pay	23,552 (7.5)	435 (4.8)	
Other	9,442 (3.0)	220 (2.4)	
Missing	8,481 (2.7)	234 (2.6)	
Mechanism of injury, n (%)			< 0.001
Fall	172,203 (55.2)	6,031 (66.0)	
Motor vehicle collision	80,637 (25.8)	1,994 (21.8)	
Motorcycle crash	13,942 (4.5)	263 (2.9)	
Pedestrian/cyclist struck	9,245 (3.0)	137 (1.5)	
Machinery	802 (0.3)	33 (0.4)	
Other	34,609 (11.1)	672 (7.4)	
Missing	779 (0.2)	8 (0.1)	

AIS, Abbreviated injury severity score; GCS, Glasgow Coma Scale

A total of 4,839 patients, or 1.5% of the overall cohort, had an unplanned ICU admission and 2,767, or 0.9% of the overall cohort, underwent an unplanned intubation. Among all patients with rib fractures managed conservatively, complications were recorded in 9,138 cases, representing 2.8% of the population. Cardiovascular complications included myocardial infarction in 350 patients (0.1%), and cardiac arrest necessitating CPR in 1,082 patients (0.3%). Stroke occurred in 321 instances (0.1%). Pneumonia was reported in 1,284 individuals (0.4%), and acute respiratory distress syndrome (ARDS) in 654 patients (0.2%). For thromboembolic events, deep vein thrombosis (DVT) was identified in 664 patients (0.2%), and pulmonary embolism (PE) was recorded in 406 patients (0.1%) (Table 3).

**Table 3:** Crude outcomes in conservatively managed rib fracture patients

	All patients (*N* = 321,355)
In-hospital mortality, n (%)	5,266 (1.6)
Any complication, n (%)	9,138 (2.8)
Myocardial infarction, n (%)	350 (0.1)
Cardiac arrest with CPR, n (%)	1,082 (0.3)
Stroke, n (%)	321 (0.1)
DVT, n (%)	664 (0.2)
Pulmonary embolism, n (%)	406 (0.1)
ARDS, n (%)	654 (0.2)
Pneumonia, n (%)	1,284 (0.4)
Unplanned intubation, n (%)	2,767 (0.9)
Unplanned admission to the ICU, n (%)	4,839 (1.5)

DVT, Deep vein thrombosis; ARDS, Acute respiratory distress syndrome; ICU, Intensive care unit

**Fig. 1:**
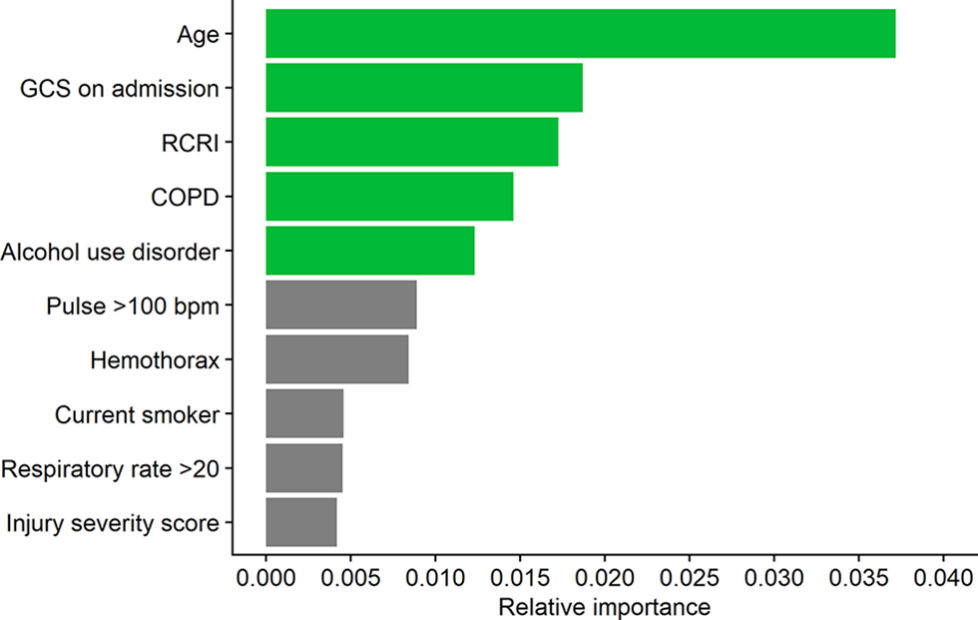
Top ten predictors of complications in all rib fracture patients. GCS, Glasgow Coma Scale; RCRI, Revised Cardiac Risk Index; COPD, Chronic obstructive pulmonary disease

Based on the variables’ PI for predicting in-hospital complications, the top five predictors for in-hospital complications were age, GCS on admission, the RCRI, chronic obstructive pulmonary disease, and alcohol use disorder (Fig. 1). The LR model using these predictors was able to attain an acceptable discriminative ability for complications [AUC (95% CI): 0.72 (0.71–0.72)], according to the cutoffs described by Hosmer and Lemeshow [28]. 

Among isolated rib fracture patients, those who suffered a complication tended to be older (72 vs. 67 years old, *p* < 0.001), frailer (OFS ≥ 2: 11.5% vs. 6.9%, *p* < 0.001), and suffer from an elevated cardiac risk (RCRI ≥ 2: 16.2% vs. 6.1%, *p* < 0.001) (Supplemental Table 1). Those who suffered a complication were also more severely injured (Thorax AIS ≥ 3: 65.1% vs. 54.9%, *p* < 0.001) and more likely to be hypotensive (4.1% vs. 1.3%, *p* < 0.001), tachycardic (25.0% vs. 16.2%, *p* < 0.001), desaturated (9.1% vs. 3.0%, *p* < 0.001), tachypneic (24.3 vs. 16.1, *p* < 0.001), as well as unconscious at admission (6.7% vs. 1.2%, *p* < 0.001) (Supplemental Table 2). Of the 183,303 isolated rib fracture patients, 4,021 patients (2.2%) suffered a complication, with 1.2% having an unplanned admission to the ICU and 0.6% an unplanned intubation (Supplemental Table 3).

**Fig. 2:**
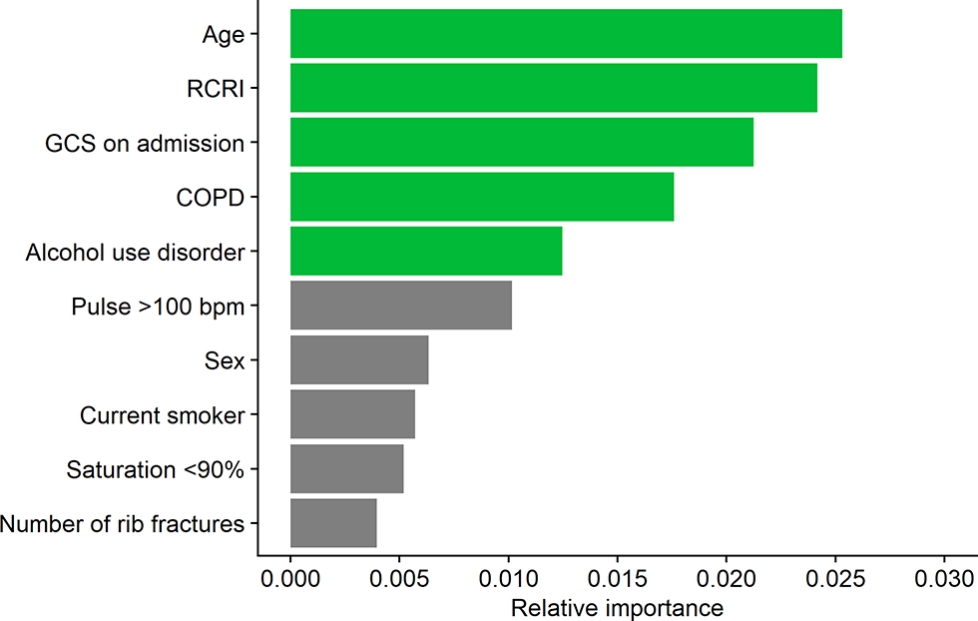
Top ten predictors of complications in isolated rib fracture patients. RCRI, Revised Cardiac Risk Index; GCS, Glasgow Coma Scale; COPD, Chronic obstructive pulmonary disease

The top five predictors of complications in isolated rib fracture patients were the same as in the overall cohort; however, the order of importance was instead age, the RCRI, GCS on admission, chronic obstructive pulmonary disease, and alcohol use disorder (Fig. 2). The LR model based on these predictors was also able to attain an acceptable discriminative ability [AUC (95% CI): 0.72 (0.71–0.73)] (Table 4) [28]. 

**Table 4:** Predictive ability of model using the top 5 most important variables for predicting complications

Cohort	AUC (95% CI)	Sensitivity (95% CI)	Specificity (95% CI)	Accuracy (95% CI)
All rib fractures (*N* = 321,355)	0.72 (0.71–0.72)	0.63 (0.62–0.64)	0.69 (0.68–0.70)	0.69 (0.69–0.69)
Isolated rib fractures (*N* = 183,303)	0.72 (0.71–0.73)	0.67 (0.66–0.69)	0.65 (0.64–0.67)	0.65 (0.65–0.65)

The models for complications use age, Glasgow Coma Scale on admission, the Revised Cardiac Risk Index, chronic obstructive pulmonary disease, and alcohol use disorder as predictors

## Discussion

In the current analysis, consisting of over 320,000 conservatively managed rib fracture patients, it was found that the top five predictors of in-hospital complications consist of age, GCS on admission, the RCRI, chronic obstructive pulmonary disease, and alcohol use disorder. These variables alone were sufficient for achieving an acceptable predictive ability in rib fracture patients, both those with and without concomitant intrathoracic injuries. These results underscore the importance of cardiovascular risk, age, and comorbidity in predicting complications, emphasizing their potential utility in informing targeted interventions and personalized care strategies for patients with rib fractures.

The current findings align with previous published data in regard to associated factors for adverse outcomes. Barry et al. conducted an analysis involving 255 geriatric patients with rib fractures, revealing a significant correlation between a GCS below 15 and an increased likelihood of ICU admission, upon univariate analysis [14]. Not surprisingly, age was the most important risk factor for in-hospital complication, closely followed by cardiovascular risk in patients suffering isolated rib fractures. In a study examining 148 cases of isolated rib fractures, diabetes mellitus, a component of the RCRI, emerged as a noteworthy factor linked to heightened morbidity [15]. Additionally, Kishawi et al. identified the significance of congestive heart failure, another component of the RCRI, as a risk factor for adverse outcomes in adult patients with singular rib fractures [16]. Finally, the development and validation of the Rib Fracture Frailty Index highlighted the importance of congestive heart failure and chronic kidney failure, both components of the RCRI which were ranked the 2nd and 3rd most important measures of frailty [13], and by extension significantly associated with morbidity [29–31]. 

These results highlight the substantial reliance on preadmission factors in predicting rib fracture patient morbidity, with older individuals as well as those with preexisting comorbidities and an elevated cardiovascular risk being at a disproportionate risk of further deterioration after admission. It is also worth noting that while the injury severity score was among the top ten predictors of complications, it appears to play a less significant role than the aforementioned factors when surgical intervention is not indicated or carried out. Understanding these risk profiles allows healthcare providers to proactively address the unique needs of this population, potentially mitigating complications through targeted interventions and specialized care. This also suggests that effective interventions and tailored care plans should be established early in the treatment process, recognizing the influence of patients’ preexisting health states on outcomes.

A crucial component is the prompt identification of patients necessitating intensive care. The present analysis indicates that elderly patients and individuals with substantial cardiovascular comorbidities face an elevated risk of complications and subsequent decompensation. A study by Bowman et al. observed that increased hospital-level ICU admissions led to improved outcomes for older patients with isolated rib fractures [32]. Furthermore, a prior investigation of elderly individuals with acute chest trauma by Pyke et al. indicated that immediate ICU admission correlated with enhanced outcomes [33]. Moreover, prior papers have found that geriatric ICU trauma patients exhibited lower mortality rates than could be expected based on the severity of their injuries [34, 35]. Conversely, other studies recommend against direct admission of geriatric patients to the ICU. For example, Naar et al. found that most elderly patients with isolated rib fractures may be safely handled in the ward, with a low risk of ward failure [36]. Additionally, a separate investigation by Bowman et al., studying isolated rib fracture patients that were > 50 year old, discovered that 63% of patients were admitted to the ICU, yet only 33% of these ICU-admission patients received critical care interventions or suffered events necessitating critical care [37]. 

In order to mitigate the risk of complications in patients with rib fractures, various additional proactive interventions should be considered, such as enhanced monitoring, physiotherapy, and tailored pain management strategies [38, 39]. However, due to limited resources, implementing these interventions in all rib fracture patients may not be feasible. In the current investigation, the RCRI was found to be among the top predictors of in-hospital complications and has also previously been found to be able to stratify rib fracture patients based on their risk of morbidity and mortality [8]. It could therefore be a useful tool for identifying patients who are disproportionately at risk of further decompensation. However this is not the only alternative, several other risk stratification tools have also been proposed in rib fracture patients including the RibScore [12], Rib Fracture Frailty Index [13], Pain Inspiratory Effort Cough Score [4], and Orthopedic Frailty Score [9, 18]. By methodically identifying high-risk individuals, healthcare teams can more efficiently deploy resources to those who are most in need. Given the RCRI’s significance in predicting complications, it may also be worthwhile to investigate whether beta-blocker therapy could improve outcomes in this population. Beta-blockers have been linked to better outcomes in trauma patients, possibly by mitigating the hyperadrenergic response to injury [40–46]. Among hip fracture patients, this benefit appears even more pronounced in patients with an elevated RCRI as well as those who are more frail [45, 46]. 

Among geriatric and frail patients, it might also be worth investigating the benefit of patient management by multidisciplinary teams, especially through collaboration with geriatricians, akin to the established practice of orthogeriatric care for hip fracture patients [47–51]. This model could allow for care tailored to the distinct requirements of elderly patients, who frequently suffer from an elevated comorbidity burden and reduced physiological reserves [8, 9]. Previous successes in managing hip fractures using this interdisciplinary strategy highlight its potential benefits, presenting a strong argument for investigating the potential benefits of its use in the management of rib fracture patients. Several systematic reviews have found that this care model constitutes a cost-effective strategy for reducing the risk of delirium, mortality, and length of stay among hip fracture patients of whom majority are elderly frail individuals with several comorbidities [47, 48, 50]. 

This study benefits from several notable strengths, comprising one of the largest investigations in its field. The leveraging of the TQIP dataset, a national database contributed to by over 900 centers, underscores the study’s extensive reach and the diversity of data sources, enhancing the generalizability of its findings [52]. However, this study is not without its limitations. While the utilization of a national database contributes to the study’s breadth, the inherent constraints of a retrospective design also introduces further challenges. Notably, the potential for selection bias is acknowledged, as the study relies on preexisting data. Furthermore, the risk of non-differential misclassification, stemming from errors in registration, may affect the accuracy of the findings. The reliance on available variables in the dataset means certain clinical characteristics, such as pain, cough, and inspiratory ability, were not accounted for. Notably, these unmeasured variables have previously demonstrated a predictive ability for ICU admission and the need for epidural analgesia in rib fracture patients [4]. We are also not able to gauge the severity of any of the preexisting comorbidities as these conditions are coded as binary variables in TQIP. The absence of data on readmission, post-discharge complications, and specific details regarding analgesics further limits the understanding of the patient outcomes beyond the initial hospitalization period. Finally, it is important to emphasize that this study is focused on prediction rather than establishing causal relationships. This distinction emphasizes the need for caution when interpreting the results, as claims of causality cannot be made.

## Conclusion

The primary predictors of complications in conservatively managed rib fracture patients consist of cardiovascular risk, age, and level of consciousness on admission. While the overall rate of complications is low in this cohort, particular care should be taken with those who are elderly with multiple cardiovascular risk factors as they suffer from a disproportionate risk of deterioration.

## Electronic supplementary material

Below is the link to the electronic supplementary material.

Supplementary Material 1

## Data Availability

Data is provided within the manuscript or supplementary information files.

## Declarations

### Congress presentation

This work was presented at the 23rd European Congress of Trauma and Emergency Surgery in Lisbon, Portugal (28–30 April 2024).
