# Supplementary material for: Morbidity prediction in conservatively managed rib fracture patients
Source: Eur J Trauma Emerg Surg. 2025 Apr 29;51(1):184. doi: 10.1007/s00068-025-02860-4 (PMC12041140; doi:10.1007/s00068-025-02860-4)
Supplement: Supplementary file 1 — Supplementary Material 1 [file 68_2025_2860_MOESM1_ESM.docx]

| **Supplemental Table 1.** Demographics of conservatively managed isolated rib fracture patients | | | |
| --- | --- | --- | --- |
|  | **No complication (N = 179,282)** | **Any complication (N = 4,021)** | **P-value** |
| Age, median [IQR] | 67 [55-79] | 72 [62-80] | <0.001 |
| Sex, n (%) |  |  | <0.001 |
| Female | 73,355 (40.9) | 1,385 (34.4) |  |
| Male | 105,745 (59.0) | 2,633 (65.5) |  |
| Missing | 182 (0.1) | 3 (0.1) |  |
| Race, n (%) |  |  | <0.001 |
| Non-Hispanic White | 130,235 (72.6) | 3,118 (77.5) |  |
| Hispanic or Latino | 7,004 (3.9) | 86 (2.1) |  |
| Black | 14,306 (8.0) | 281 (7.0) |  |
| Other | 16,312 (9.1) | 272 (6.8) |  |
| Missing | 11,425 (6.4) | 264 (6.6) |  |
| RCRI, n (%) |  |  | <0.001 |
| 0 | 124,943 (69.7) | 2,075 (51.6) |  |
| 1 | 43,451 (24.2) | 1,291 (32.1) |  |
| 2 | 9,136 (5.1) | 504 (12.5) |  |
| 3 | 1,583 (0.9) | 130 (3.2) |  |
| ≥4 | 169 (0.1) | 21 (0.5) |  |
| OFS, n (%) |  |  | <0.001 |
| Non-frail (OFS 0) | 133,972 (74.7) | 2,411 (60.0) |  |
| Pre-frail (OFS 1) | 33,021 (18.4) | 1,148 (28.6) |  |
| Frail (OFS ≥2) | 12,289 (6.9) | 462 (11.5) |  |
| Hypertension, n (%) | 93,154 (52.0) | 2,650 (65.9) | <0.001 |
| History of angina, n (%) | 463 (0.3) | 21 (0.5) | 0.004 |
| Previous myocardial infarction, n (%) | 2,542 (1.4) | 121 (3.0) | <0.001 |
| Congestive heart failure, n (%) | 12,116 (6.8) | 662 (16.5) | <0.001 |
| History of peripheral vascular disease, n (%) | 2,357 (1.3) | 127 (3.2) | <0.001 |
| Cerebrovascular disease, n (%) | 7,012 (3.9) | 277 (6.9) | <0.001 |
| Dementia, n (%) | 10,702 (6.0) | 307 (7.6) | <0.001 |
| Non-independent functional status, n (%) | 19,445 (10.8) | 727 (18.1) | <0.001 |
| Institutionalized, n (%) | 5,295 (3.0) | 149 (3.7) | 0.006 |
| Currently receiving chemotherapy for cancer, n (%) | 1,085 (0.6) | 37 (0.9) | 0.018 |
| Metastatic cancer, n (%) | 1,685 (0.9) | 62 (1.5) | <0.001 |
| History of malignancy, n (%) | 2,654 (1.5) | 91 (2.3) | <0.001 |
| COPD, n (%) | 23,751 (13.2) | 1,224 (30.4) | <0.001 |
| Current smoker, n (%) | 33,247 (18.5) | 990 (24.6) | <0.001 |
| Chronic renal failure, n (%) | 4,248 (2.4) | 247 (6.1) | <0.001 |
| Diabetes mellitus, n (%) | 40,454 (22.6) | 1,218 (30.3) | <0.001 |
| Cirrhosis, n (%) | 3,231 (1.8) | 176 (4.4) | <0.001 |
| Coagulopathy, n (%) | 8,081 (4.5) | 320 (8.0) | <0.001 |
| Drug use disorder, n (%) | 8,164 (4.6) | 234 (5.8) | <0.001 |
| Alcohol use disorder, n (%) | 14,272 (8.0) | 638 (15.9) | <0.001 |
| Major psychiatric illness, n (%) | 22,206 (12.4) | 600 (14.9) | <0.001 |
| Advanced directive limiting care, n (%) | 6,856 (3.8) | 302 (7.5) | <0.001 |
| *RCRI, Revised Cardiac Risk Index; OFS, Orthopedic Frailty Score; COPD, chronic obstructive pulmonary disease* | | | |

| **Supplemental Table 2.** Clinical characteristics of conservatively managed isolated rib fracture patients | | | |
| --- | --- | --- | --- |
|  | **No complication (N = 179,282)** | **Any complication (N = 4,021)** | **P-value** |
| Injury Severity Score, median [IQR] | 9.0 [4.0-10] | 9.0 [5.0-10] | <0.001 |
| Head AIS, n (%) |  |  | 0.384 |
| Injury not present | 157,445 (87.8) | 3,550 (88.3) |  |
| 1 | 21,837 (12.2) | 471 (11.7) |  |
| Face AIS, n (%) |  |  | 0.453 |
| Injury not present | 152,867 (85.3) | 3,411 (84.8) |  |
| 1 | 26,415 (14.7) | 610 (15.2) |  |
| Neck AIS, n (%) |  |  | 0.754 |
| Injury not present | 177,430 (99.0) | 3,982 (99.0) |  |
| 1 | 1,852 (1.0) | 39 (1.0) |  |
| Spine AIS, n (%) |  |  | 0.018 |
| Injury not present | 177,019 (98.7) | 3,987 (99.2) |  |
| 1 | 2,263 (1.3) | 34 (0.8) |  |
| Thorax AIS, n (%) |  |  | <0.001 |
| 1 | 31,932 (17.8) | 493 (12.3) |  |
| 2 | 48,866 (27.3) | 907 (22.6) |  |
| 3 | 97,393 (54.3) | 2,541 (63.2) |  |
| 4 | 914 (0.5) | 54 (1.3) |  |
| 5 | 177 (0.1) | 26 (0.6) |  |
| Abdomen AIS, n (%) |  |  | 0.026 |
| Injury not present | 164,261 (91.6) | 3,644 (90.6) |  |
| 1 | 15,021 (8.4) | 377 (9.4) |  |
| Upper extremity AIS, n (%) |  |  | 0.991 |
| Injury not present | 146,947 (82.0) | 3,295 (81.9) |  |
| 1 | 32,335 (18.0) | 726 (18.1) |  |
| Lower extremity AIS, n (%) |  |  | 0.393 |
| Injury not present | 150,180 (83.8) | 3,389 (84.3) |  |
| 1 | 29,102 (16.2) | 632 (15.7) |  |
| External/Other AIS, n (%) |  |  | 0.472 |
| Injury not present | 170,650 (95.2) | 3,817 (94.9) |  |
| 1 | 8,632 (4.8) | 204 (5.1) |  |
| Flail chest, n (%) | 2,121 (1.2) | 111 (2.8) | <0.001 |
| Sternal fracture, n (%) | 12,912 (7.2) | 315 (7.8) | 0.133 |
| Number of rib fractures, n (%) |  |  | <0.001 |
| Single | 34,120 (19.0) | 505 (12.6) |  |
| Multiple | 145,162 (81.0) | 3,516 (87.4) |  |
| Method of analgesia, n (%) |  |  |  |
| Regional | 2,201 (1.2) | 115 (2.9) | <0.001 |
| Epidural | 702 (0.4) | 51 (1.3) | <0.001 |
| Spinal | 698 (0.4) | 56 (1.4) | <0.001 |
| Steroid use, n (%) | 3,006 (1.7) | 160 (4.0) | <0.001 |
| Anticoagulant therapy, n (%) | 877 (0.5) | 10 (0.2) | 0.043 |
| Missing | 26,603 (14.8) | 632 (15.7) |  |
| Shock index, median [IQR] | 0.58 [0.48-0.70] | 0.62 [0.50-0.77] | <0.001 |
| Missing, n (%) | 5328 (3.0) | 167 (4.2) |  |
| Systolic blood pressure <90 mmHg, n (%) | 2,330 (1.3) | 163 (4.1) | <0.001 |
| Missing | 4,366 (2.4) | 123 (3.1) |  |
| Pulse rate > 100 bpm, n (%) | 29,119 (16.2) | 1,007 (25.0) | <0.001 |
| Missing | 4,078 (2.3) | 105 (2.6) |  |
| Temperature <35°C, n (%) | 736 (0.4) | 68 (1.7) | <0.001 |
| Missing | 13,722 (7.7) | 396 (9.8) |  |
| Temperature ≥38°C, n (%) | 1,235 (0.7) | 72 (1.8) | <0.001 |
| Missing | 13,722 (7.7) | 396 (9.8) |  |
| Saturation <90%, n (%) | 5,294 (3.0) | 367 (9.1) | <0.001 |
| Missing | 8,186 (4.6) | 222 (5.5) |  |
| Respiratory rate >20, n (%) | 28,929 (16.1) | 976 (24.3) | <0.001 |
| Missing | 5,385 (3.0) | 164 (4.1) |  |
| Respiratory rate <12, n (%) | 1,700 (0.9) | 75 (1.9) | <0.001 |
| Missing | 5,385 (3.0) | 164 (4.1) |  |
| GCS on admission, n (%) |  |  | <0.001 |
| Mild (GCS 14-15) | 162,253 (90.5) | 3,315 (82.4) |  |
| Moderate (GCS 9-13) | 3,061 (1.7) | 157 (3.9) |  |
| Severe (GCS 3-8) | 2,146 (1.2) | 270 (6.7) |  |
| Missing | 11,822 (6.6) | 279 (6.9) |  |
| Trauma center level, n (%) |  |  | <0.001 |
| Level 1 | 65,649 (36.6) | 1,667 (41.5) |  |
| Level 2 | 45,552 (25.4) | 940 (23.4) |  |
| Level 3 | 13,589 (7.6) | 228 (5.7) |  |
| Not verified/designated | 54,492 (30.4) | 1,186 (29.5) |  |
| Insurance, n (%) |  |  | <0.001 |
| Commercial insurance | 55,293 (30.8) | 916 (22.8) |  |
| Non-commercial insurance | 102,625 (57.2) | 2,800 (69.6) |  |
| Self-pay | 11,307 (6.3) | 127 (3.2) |  |
| Other | 5,320 (3.0) | 86 (2.1) |  |
| Missing | 4,737 (2.6) | 92 (2.3) |  |
| Mechanism of injury, n (%) |  |  | <0.001 |
| Fall | 104,002 (58.0) | 2,847 (70.8) |  |
| Motor vehicle collision | 50,852 (28.4) | 860 (21.4) |  |
| Motorcycle crash | 5,988 (3.3) | 57 (1.4) |  |
| Pedestrian/cyclist struck | 3,559 (2.0) | 32 (0.8) |  |
| Machinery | 418 (0.2) | 9 (0.2) |  |
| Other | 14,091 (7.9) | 212 (5.3) |  |
| Missing | 372 (0.2) | 4 (0.1) |  |
| *AIS, Abbreviated injury severity score; GCS, Glasgow Coma Scale* | | | |

| **Supplemental Table 3.** Crude outcomes in conservatively managed isolated rib fracture patients | |
| --- | --- |
|  | **All patients (N = 183,303)** |
| In-hospital mortality, n (%) | 2,580 (1.4) |
| Any complication, n (%) | 4,021 (2.2) |
| Myocardial infarction, n (%) | 181 (0.1) |
| Cardiac arrest with CPR, n (%) | 487 (0.3) |
| Stroke, n (%) | 170 (0.1) |
| DVT, n (%) | 289 (0.2) |
| Pulmonary embolism, n (%) | 171 (0.1) |
| ARDS, n (%) | 248 (0.1) |
| Pneumonia, n (%) | 529 (0.3) |
| Unplanned intubation, n (%) | 1,104 (0.6) |
| Unplanned admission to the ICU, n (%) | 2,189 (1.2) |
| *DVT, Deep vein thrombosis; ARDS, Acute respiratory distress syndrome; ICU, Intensive care unit* | |
